# Supplementary material for: Evidence for a universal association of auditory roughness with musical stability
Source: PLoS One. 2023 Sep 20;18(9):e0291642. doi: 10.1371/journal.pone.0291642 (PMC10511120; doi:10.1371/journal.pone.0291642)
Supplement: S1 Appendix — (PDF) [file pone.0291642.s001.pdf]

Appendix for 'Evidence for a universal association of auditory roughness with musical stability'

Western music

We define Western music as any music that makes substantial use of a set of structural features commonly found in European music from at least the 17th century (although some of these date substantially further back in the European tradition; some, somewhat later). These include the use of:

- instruments (and human voice) that produce harmonic complex tones and, hence, induce clearly perceptible pitches;
- a set of discrete pitches tuned to a meantone-like system, the most common of which, in contemporary practice, is 12-tone equal temperament where every octave is divided into 12 equal semitones (a meantone system contains intervals (octaves) with a frequency ratio close to 2/1, and intervals (perfect fifths) with frequency ratios close to, or slightly smaller than, 3/2, which ensures that 4 perfect fifths minus 2 octaves approximates a frequency ratio of 5/4);
- frequent use of the diatonic scale (a *well-formed scale* [1] with 5 large steps and 2 small, where the large steps are approximately twice the size of the small);
- frequent use of major and minor chords and, sometimes, diminished and augmented chords, and their extensions (sevenths, ninths, etc.);
- *modulations* between diatonic scales, which are typically smooth because the two scales will share many common pitches and are typically mediated via a pivot chord that is common to both scales;
- common assertion of a tonic pitch class or tonic major or minor chord through the use of cadences, which are well-established chord progressions that typically involve movement from a dominant seventh chord (or major chord) to a major or minor chord a perfect fifth below and, often, this dominant chord is preceded by a chord containing the scale's fourth degree (the subdominant);
- an isochronous hierarchical binary or ternary metrical structure, whereby the fastest metrical level (rhythmic pulse) is grouped into either twos or threes to make a slower metrical level, which is itself grouped into twos or threes to make an even slower metrical level, and so on.

This definition of Western music is, therefore, one that allows for Western music or *Western-like music* to be produced in non-Western countries. For example, the Western-like guitar band music in PNG is strongly informed by Western music (through historical musical training provided by missionaries and the use of Western musical instruments [2]) and comprises almost all of the characteristics of Western music (as defined above), whilst still being quite distinct and recognizable as a genre or style of music that is different from anything actually produced in the West. Of course, this definition of Western music may seem flawed. For example, twentieth century atonal (including most serial) music would not fulfill all of the above criteria and, yet, is clearly a Western phenomenon. However, it is the term 'Western music' that is problematic here, rather than the definition provided above; unfortunately, no other English term is

available that can capture all and only the set of features above (for example, the term 'common practice' refers to a specific historical period and excludes 20th- and 21st-century pop, jazz, and film music); furthermore, we feel that in most readers' minds 'Western music' will most readily evoke the characteristics listed above, and this motivates our choice of a practical, though imperfect, term.

Background information on the Uruwa River valley

General background

The Uruwa River valley is a remote twelve-village cloud forest area in the Saruwaged Mountains, Morobe Province, Papua New Guinea. The area is accessible only by small plane or, for locals, a difficult multi-day hike. Elevation reaches peaks of 4,000 m in the surrounding mountains. As described by [3], there is hardly any level ground and people live, move and cultivate their crops on steep slopes. People are self-sufficient and expert farmers who live mostly without electricity. The area has had mobile phone coverage since mid-2015, but internet access on this network is practically impossible. The speech varieties of the villages constitute a dialect continuum [3].

As is common in such remote mountain areas of PNG, there are no roads to the area or nearby mountain regions. The Uruwa River Valley is accessible to outsiders only by small airplane; in the six-village southern, higher-elevation part of the river valley, such airplanes must land on an inclined grassy airstrip at Yawan village that was cleared by villagers, using hand tools, over several years in the 1970s, then extended in the 1990s. Historically, the village communities of the Uruwa River Valley are said to have lived in a state of uneasy truce with each other, punctuated by conflicts. This is reflected in the locations of the villages – each is separated from the others by geographic barriers, such as waterways. Each village community comprises two or more clan groups.

Most early missionary activity in the Uruwa River Valley was done by Papua New Guinean missionaries who lived in the region for many years, and also introduced coffee farming, cabbage, pumpkin, peanuts, and some other crops. By the 1960s, most people in the area had been baptized as Lutheran Christians. The Lutheran church used another Papuan language, Kâte, as a lingua franca in much of northeast New Guinea, and some older Nungon speakers attended a Kâte school and became literate in Kâte. Songs in the Kâte language are still known by Nungon speakers, and performed occasionally in the Lutheran churches in Worin and Mup. The Lutheran church was known for welcoming local musical traditions and encouraging the use of *uwing* drums and local languages in services. With the advent of the *stringben* style, that also became incorporated into Lutheran services. In contrast, the later-arriving Seventh-Day Adventist church, which made inroads in the Uruwa area from the late 1970s on, strictly prohibited use of any PNG music styles in church services: hymns must be drawn from the official *SDA Hymnal*, and sung to traditional North American and European melodies. People baptized into the SDA church had to renounce playing the *uwing* drum, in addition to the major lifestyle shifts required of SDA adherents: abstention from consuming pork (traditionally, central to feasts and gatherings in much of PNG), tobacco and betelnut. Today, Towet village is the only Uruwa village in which the majority of people adhere to the SDA church. In all other villages, SDA followers are either in the minority or non-existent. Lutheran churches today are found in Worin and Mup villages, and SDA churches are found in Towet, Yawan, and Worin villages. There used to be a Lutheran church in Kotet, which was demolished at the end of 2011.

A major cultural shift began in 1995 in the southern Uruwa villages, when Towet man Dono Ögate and his wife Eni, who had married into the area from the Nukna region to the east, returned to the region from the port city of Lae and began a

concerted effort to 'develop' their community. Eni trained as the founding teacher of the first elementary school in Yawan village, established in 1998, and together the couple began distributing non-traditional clothing, such as T-shirts and shorts, to their community, and teaching them to speak and read the English-based creole Tok Pisin. In 2019, Dono Ögate was recognized for this work by the Digicel Foundation: he received the national 2019 Overall Man of Honour award.

Musical background

There are two main strands of musical traditions in the Uruwa region, as elsewhere in the region [4]. Older traditional indigenous genres are accompanied by hourglass-shaped hand-drums (called *uwing* in Nungon) or by flute. Since the 1970s, a style of Western-influenced sung genre, called *stringben* in Tok Pisin (from *string band*), and characterized by guitar or ukulele accompaniment, has co-existed with the older musical styles [5, 6]. Music is primarily heard in weekly church services, on special occasions, or when individuals sing while going about daily activities, or practice for performances. There are no professional musicians, nor people who specialize in musical performance; traditionally, all women sang and danced in communal gatherings, and all men sang, danced, and played *uwing*. Elementary-age children may learn to sing songs in the local language, Nungon, or in Tok Pisin, at school. Few people in the area own radios or other music players, and there is no mains electricity source for charging mobile electronic devices. There is no equipment for viewing movies or videos in the area; nor, for that matter, are any of the Uruwa people who live in distant diaspora areas known to own televisions.

Participants, data collection and exclusions

Table A1. Test locations and participant numbers.

Dates	Test location	Participants' origin	No. Participants	Experimenters
27 June–7 July 2019	Towet	Towet	87	E. A. Smit, A. J. Milne
27 June–7 July 2019	Towet	Worin	1	E. A. Smit, A. J. Milne
14–25 July 2019	Mup	Mup	18	B. Waum, N. Urung
14–25 July 2019	Mup	NA (missing interview)	1	B. Waum, N. Urung
30 July–4 August 2019	Mitmit	Mitmit	1	B. Waum, N. Urung, N. Ögate
30 July–4 August 2019	Mitmit	Bembe	19	B. Waum, N. Urung, N. Ögate
30 July–4 August 2019	Mitmit	Worin	3	B. Waum, N. Urung, N. Ögate
6–8 August 2019	Kotet	Kotet	15	B. Waum, N. Urung, N. Ögate
1–3 October 2019	Kotet	Kotet	16	B. Waum, N. Urung, N. Ögate
3–8 October 2019	Yawan	Yawan	9	B. Waum, N. Urung, N. Ögate

Table A2. Before and after exclusions due to patterned responses, the numbers of dyad and triad blocks, and participants partaking of each or either. Each participant was presented with two blocks of dyads and one block of triads.

Group	Block	No. blocks		No. participants	
		Before	After	Before	After
Uruwa: all	Dyads	338	246	169	138
	Triads	169	123	169	123
	Either			169	144
Uruwa: minimal	Dyads	58	34	29	19
	Triads	29	21	29	21
	Either			29	22
Uruwa: Lutheran	Dyads	88	72	44	39
	Triads	44	36	44	36
	Either			44	39
Uruwa: SDA	Dyads	192	140	96	80
	Triads	96	66	96	66
	Either			96	83
Sydney: all	Dyads	158	158	79	79
	Triads	79	78	79	78
	Either			79	79
Sydney: non-musician	Dyads	120	120	60	60
	Triads	60	59	60	59
	Either			60	60
Sydney: musician	Dyads	38	38	19	19
	Triads	19	19	19	19
	Either			19	19

Stimuli

Intervals

Every ordered pair of intervals comprised one interval from the set $\{0, 1, 2, \ldots, 12\}$ (these are interval widths in semitones) and another larger interval obtained by one of the following five transformations (in semitones) of the lower and upper pitches: $(0, 1), (-1, 0), (-1, 1), (-1, 2), (-2, 1)$. For instance, applying the transformation $(-1, 2)$ to the perfect fifth C–G (which has a width of 7) produces the minor seventh B–A (which has a width of 10). Disregarding transposition, this results in $5 \times 13 = 65$ pairs of intervals, hence 130 ordered pairs. Participants were divided into four blocks and heard a subset of 60 of these ordered intervals, in each case followed by the same interval pair in reverse order. Each such pair of ordered pairs was randomly transposed by less than an octave. Across the experiment, interval pairs where one interval was either a unison or an octave were tested half as often as every other interval pair.

Triads

The triad pairs used in the experiment are detailed in Fig A1.

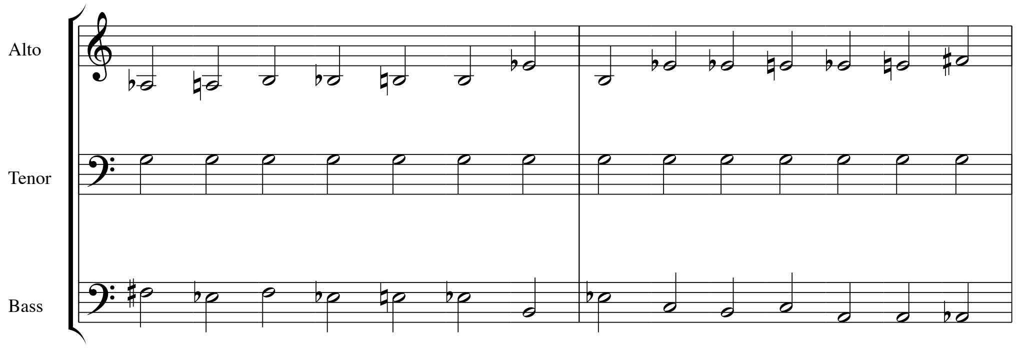

Fig A1. Triad pairs used in the experiment. In their triad block, each participant either heard all 42 ordered pairs of the first seven chords, or all 42 ordered pairs of the second seven chords. Two of the chords are in both groups, so there is a grand total of 82 distinct ordered pairs. Five out of the second set of seven are – by Western music-theoretical characterizations – open-voiced versions of five from the first set of seven. These open and closed versions are sometimes in a different inversion (e.g., the semitone cluster); sometimes in the same inversion (e.g., the major and minor chords are always in root position). Each trial comprised one ordered pair followed by the same pair in reversed order. Each such pair of ordered pairs was randomly transposed by less than an octave. Note that each accidental applies only to the note it immediately precedes.

Ethical considerations and community relations

This study was approved by the WSU Human Ethics Committee (H13179). The PNG National Research Institute granted research visas to A.J.M. and E.M.S. based on positive evaluation of this project. Beyond these official approvals, the Towet village community went to extraordinary lengths to show that they approved of the research and welcomed the researchers.

Months in advance of the foreign researchers' arrival into the Uruwa area, local organizers Stanly Girip, James Jio and Lyn Ögate were already corresponding with H.S.S. to plan the programs for the opening and closing ceremonies of the researchers' field trip, which the three of them typed up and distributed, using a portable printer run on solar power. The entire thirty-household Towet community stockpiled communal stores of firewood, farm vegetables, and chickens for months to ensure that they would be available to serve as participants, cooks, security, and research assistants during the researchers' stay in Towet [7, 8].

When the researchers arrived, the whole Towet community took two weeks off from their usual hard daily labour of subsistence agriculture, hunting, and foraging. This was exceptional; H.S.S. has never observed or heard of such a long period of abstinence from subsistence activities by an entire village in the past ten years, at least. Instead of pursuing these activities, the village subsisted on the enormous amounts of foodstuffs and firewood they had carefully accrued over many months, in anticipation of the researchers' visit.

For the research team, respect for local culture and mores, and respect for the local language, were paramount. While still overseas, H.S.S. prepared the rest of the research team for the trip through a written document, and meetings detailing special cultural considerations, and explaining key words in the Nungon language. Official ethics approvals do not often take into account the effect of use of a lingua franca by outsiders on the prestige of a small, local language. We planned our project to evince respect, not just for local culture and traditions, but also for the local language, Nungon. All researchers used Nungon for basic greetings and niceties, and Nungon was the medium of experimentation.

All aspects of the research trip were managed by S. Girip, J. Jio, and L. Ögate, in conjunction with H.S.S. These three local organizers handled recruitment of experiment participants, cooks, security, and research assistants, but confirmed at each step of the way with H.S.S. what they were doing, and why. A major principle for them was ensuring equitable distribution of paid work among Towet community members, and especially distributing work equally among Towet's three major clans. The community also hosted a non-Towet man who was fleeing violence in a nearby village, and he was given paid work on the security detail as a gesture of welcome. Experiment participation, and involvement in the three other categories of paid work, was purely voluntary. For instance, one of the other three experiments run at the same time as this one involved methods that were perceived by some community members as potentially invasive: EEG. Many fewer community members volunteered for the EEG study than for the present study.

Throughout the trip, H.S.S. and the three community organizers, as well as other local leaders, such as two teachers and a former government Councillor, kept close tabs on community sentiment, and checked in with each other regularly. H.S.S. served as translator and mediator between the other researchers and the organizers. The second Saturday afternoon, H.S.S. led the research team on a meet-and-greet tour of the village, to allow community members who had not yet directly interacted with the outside researchers to share their own stories with the outsiders. This was a moving experience for the outside researchers, who were thus able to meet community members with physical and mental disabilities who had not participated in the experiments.

Community leaders, including the teachers and former government Councillor, participated in the experiments as desired, and also gave speeches at the opening and closing ceremonies of the field trip. In these ceremonies, members of the community overwhelmingly expressed warm feelings toward the researchers and their work, and welcomed them back any time they would be able to return.

Uruwa interview

The interview with participants from the Uruwa River Valley consisted of the following questions with possible answers provided in brackets:

1. What is your age?
2. Which village are you from?
3. Male/female?
4. Do you go to church? (Yes; no; sometimes)
5. Which do you go to? (SDA; Lutheran; I do not go to church)
6. When you go to church, do you sing songs or do you just listen? (I sing; I just listen)
7. Are you a song leader? (Yes; no)
8. Do you sing hymns outside of church? (Yes; no; sometimes)
9. When they sing songs, do you understand the words you sing?(Yes; no; sometimes)
10. Are you always happy or always sad when you hear all church songs, or are you happy when you hear some church songs and sad when you hear other songs? (Always happy; always sad; sometimes happy sometimes sad)

11. Why? Is the meaning of the words grabbing you that you feel sad or the sound? (Meaning of the words; the sound; meaning of the words and the sound)

12. Did you used to try different songs? (Yes; no)

13. What types of music did you used to play or sing? (*Biru* (a local flute); *uwing* (an hour glass shaped drum); guitar)

14. If a song seized you on the insides, how did it make you feel? (Happy; sad; sometimes happy sometimes sad)

15. When you used to sing a song in other peoples' language, would you understand? (Yes; no)

16. When you were small, how did the older people used to do songs or music? (*Biru*; *uwing*; guitar)

17. These days on your phone or radio, what songs do you find beautiful? (Open question, only answered if people have a phone or radio)

18. These days, do you listen to songs from other places or not? (Yes; no)

19. From where?

Calculation of psychoacoustic and musical features

The audio features were calculated using functions from the first author's Music Perception Toolbox (for MATLAB) (`https://github.com/andymilne/Music-Perception-Toolbox`). The script importing and processing the audio files and calling the functions is available at `https://osf.io/ux4q5/`. In every case, the predictor used in the model is the change of the respective feature from the third to the fourth chord. The resulting Δ*roughness*, Δ*harmonicity*, and Δ*spectral entropy* values were standardized (mean of 0, standard deviation of 1), the Δ*mean pitch* values were centred (mean of 0) but kept in semitone units.

Roughness

The audio of each chord (1.67 seconds long, 44.1 KHz, 16-bit) was Fourier transformed. The resulting spectrum was converted to the log-frequency domain and smoothed with a Gaussian kernel with a standard deviation of 12 cents. The smoothing is necessary to ensure that close spectral peaks that result from vibrato in the signal do not contribute to the roughness calculation (an alternative is to calculate the peaks over small windows of the audio, as in the MIR Toolbox [9]; both methods reduce the spectral resolution). From the smoothed signal, peaks were extracted and returned to the frequency domain to allow the dissonances of all partial pairs to be calculated – using Sethares' parameterization [10] of the Plomp-Levelt dissonance function [11] – and summed. For comparison, the roughness values produced by the Music Perception Toolbox (12 cent smoothing) and the MIR Toolbox (using the default 50 ms windows) have a Pearson correlation of 0.94 across the chords used in the experiment.

Harmonicity

The spectrum of each chord was converted to the log-frequency domain and smoothed with a Gaussian kernel with a standard deviation of 12 cents. The smoothing accounts for perceptual pitch uncertainty. Using the method established in [12] (and compared with other methods for calculating harmonicity in [13]), the log-frequency spectrum was cross-correlated with a similarly smoothed spectrum from a template harmonic complex tone with harmonics with amplitude $1/h$, where h is the harmonic number (this is the spectrum of a sawtooth wave). The maximum value of the cross-correlation (after appropriate normalization) gives the cosine similarity between the audio and the template factoring out transposition, and serves as a straightforward measure of harmonicity.

Spectral entropy

The spectrum of each chord was converted to the log-frequency domain and smoothed with a Gaussian kernel with a standard deviation of 12 cents. The smoothing accounts for perceptual pitch uncertainty. Using the method established in [14] and experimentally tested in [15], the smoothed log-frequency spectrum is normalized (so its integral is 1) and its normalized entropy calculated. This serves as a straightforward measure of the complexity or unpredictability of the spectrum.

Mean pitch

Mean pitch is calculated as the mean MIDI pitch of all pitches in each chord.

Modelling

The data were analysed in R [16] using the brms package [17, 18], which is a front end for the Bayesian inference and Markov chain Monte Carlo (MCMC) sampler Stan [19].

Chord-type models

The chords were classified in three different ways: disregarding timbre and chord transposition; disregarding timbre, transposition, and upper individual-pitch octave (put differently, the lowest pitch of the chord is designated 0); disregarding timbre, chord transposition, and individual-pitch octave. The first representation, therefore, classifies the chords (C4, E4, G4) and (D4, F♯4, A4) as the same because they are chord transpositions (every pitch in the second chord is a whole tone higher than every pitch in the first chord). The second representation additionally classifies the chords (C4, E4, G4), (C4, E4, G5), and (C4, E5, G6) as equivalent because one or more upper pitch differs by one or more octaves; hence (C4, E4, G4) and (D4, F♯4, A5) would also be classified as equivalent. This representation corresponds to familiar labels such as 'root-position major', 'first-inversion diminished', 'second-inversion minor', and so forth. The third representation (T_n-type) additionally classifies the chords (C4, E4, G4) and (C4, G3, E4) the same because individual pitches differ by one or more octaves (note that the second classification would treat them as different because the lowest pitch in the first and second chord have different pitch classes: C in the first, G in the second). This classification corresponds to familiar chord labels such as 'major', 'minor', 'diminished' (i.e., it ignores whether the chords are in root-position, first or second inversion), and so forth.

For each of these three classification schemes, the final two chords were coded as specified in [20] to allow for a Thurstone paired comparisons model to be run as a

straightforward Bayesian multilevel logistic regression model. In this context, a Thurstone model assumes that, relative to an arbitrarily chosen reference chord with a stability value of 0, every other chord has a latent stability value. This latent relative stability is assumed to be normally distributed and is conventionally scaled so that its units are standard deviations. The multilevel structure was specified to allow effects to vary between participant but only within each exposure group (so there is no information pooled between exposure groups). One chord type was, arbitrarily, specified as the reference – it was included as a population-level effect but with a prior fixing its value to 0 and not included as a group-level effect; this is equivalent to completely omitting this chord from the model, but including it in this way makes plotting the model's results easier (see Fig 3 in the main text, and Figs A2 and A3). Priors for all other effects on the latent scale were weakly informative normal distributions with a mean of 0 and a standard deviation of 1 in order to regularize (towards zero) any effects that may be only weakly informed by the data. The fitted models and associated R code are available at `https://osf.io/c3e9y/`.

Psychoacoustic models

Bayesian multilevel logistic regression models were fitted with all subsets of the predictors Δ*roughness*, Δ*harmonicity*, Δ*spectral entropy*, and Δ*mean pitch*. In each case, they interacted with a five-level factor *exposure group* and these all interacted with a two-level factor *trial_type* (both factors were sum-coded to facilitate obtaining main effects of the other terms). All within-participant predictors were allowed to vary between different participants within each exposure group (but not between exposure groups to avoid pooling information between exposure groups) using group-level (random) effects. The intercept, which also varies by participant, can adjust for each participant's bias in preferring to choose, for example, the second stimulus. All continuous predictors were standardized except for mean pitch difference, which was left in semitone units to aid interpretability (this predictor has a standard deviation of 0.52 semitones). All predictors' effects were given weakly informative priors (a Student's t-distribution with a mean of 0, 3 degrees of freedom, and a scale of 1) in order to regularize (towards zero) any effects that may be only weakly informed by the data. The strength of evidence for each directional hypothesis was obtained from Bayesian evidence ratios which are the posterior odds of the effect being in the direction specified in the hypothesis. For a one-sided hypothesis, an evidence ratio greater than 19 is loosely analogous to a one-sided p-value below 0.05 [21, 22]; that is, there is a posterior probability of 95% the effect is in the hypothesized direction. We also used ROPE tests to quantify the probability the effect is practically equivalent to zero. A commonly used region of practical equivalent for standardized effects in logistic regression models is the interval $[-0.18, 0.18]$ (this is analogous to $[-0.10, 0.10]$ in a linear model). However, as noted in the main text, stimuli differing by five or more standard deviations can be freely chosen by a musical composer or performer. For this reason, we choose $[-0.18/5, 0.18/5] = [-0.036, 0.036]$ as a more reasonable region of practical equivalence to zero.

Correlations of predictors

Roughness, harmonicity, and spectral entropy can be highly correlated across musical data; particularly the idealized versions of these predictors. This can be problematic because it makes it hard to assess their individual contributions. For our data, across the above three features and mean pitch, there were no problems with multicollinearity – the largest VIF (variance inflation factor) was only 2.04. (This is, in part, due to the

selection of chords used and to most of these features being calculated directly from the audio signal rather than from idealized spectra derived from the notated pitches.)

Table A3. Correlations of psychoacoustic predictors.

	Δroughness	Δharmonicity	Δspectral entropy	Δmean pitch
Δroughness	1.00	−0.27	0.54	−0.10
Δharmonicity	−0.27	1.00	−0.59	0.02
Δspectral entropy	0.54	−0.59	1.00	−0.14
Δmean pitch	−0.10	0.02	−0.14	1.00

Variable selection

Models with every subset of roughness (R), harmonicity (H), spectral entropy (E) and mean pitch (P) were compared with PSIS-LOO, which is a fast approximation of leave-one-out cross-validation [23] used to estimate each model's ability to generalize to out-of-sample data. The variables in each model are indicated by the suffix in its name, while 'mdl_int_triad_1' is an intercept-only model. Their relative performance is listed below, with the best model (with highest 'elpd_diff') at the top. The performance of the second-, third-, and fourth-listed models is not significantly different to the top-listed model because each 'elpd_diff' is less than two times the standard error of its estimated size. In this article, we report results from the top-listed 'RHP' model.

Table A4. Model comparisons using PSIS-LOO. ELPD is the theoretical expected log pointwise predictive density for a new dataset; higher ELPDs show the model generalizes better to unseen data. ΔELPD is the difference of each model's ELPD compared to the best model's ELPD. SE(ΔELPD) is the standard error of ΔELPD.

	ΔELPD	SE(ΔELPD)
mdl_int_triad_RHP	0.0	0.0
mdl_int_triad_RHEP	−6.1	5.7
mdl_int_triad_REP	−12.5	8.9
mdl_int_triad_RP	−13.9	8.9
mdl_int_triad_RH	−26.9	10.1
mdl_int_triad_RHE	−29.3	11.5
mdl_int_triad_RE	−36.9	13.2
mdl_int_triad_R	−40.6	13.2
mdl_int_triad_HEP	−121.8	18.0
mdl_int_triad_EP	−125.9	19.1
mdl_int_triad_HE	−149.4	20.5
mdl_int_triad_E	−152.8	21.5
mdl_int_triad_HP	−169.8	19.4
mdl_int_triad_H	−202.8	21.7
mdl_int_triad_P	−205.0	22.1
mdl_int_triad_1	−237.2	24.0

Summary of the 'RHP' model

The VIFs for the RHP model are all below 1.1, hence the predictors are almost fully independent for these data. The fitted model and associated R code are available at `https://osf.io/c3e9y/`.

Hypothesis tests for the 'RHP' model fitted to all data (i.e., including patterned responses)

Table A5. Hypothesis tests and summaries of the main effects (across dyads and triads) of Δ*roughness*, Δ*harmonicity*, and Δ*mean pitch*, in the five groups of participants with no data excluded. This table follows the same format as Table 1 in the main text but, here, the model has been fitted to all of the data (i.e., including the patterned responses that indicate the task instruction was not being followed). All and only hypotheses that were strongly evidenced in Table 1 in the main text are also strongly evidenced here. 'Mean', 'Q5%', and 'Q95%' are the mean and 90% equal-tailed credibility interval for the logit-scale effect of a standard deviation increase in roughness and harmonicity, and a one-semitone increase in mean pitch. 'Evid.ratio' is the odds the effect is in the direction specified by the hypothesis, while 'Post.p' is its associated posterior probability. 'ROPE' is the probability the effect is practically equivalent to zero [24], which we define as being in the interval $[-0.036, 0.036]$.

Grp by hypothesis	Mean	Q5%	Q95%	Evid.Ratio	Post.p	ROPE
Effect of Δ*roughness* < 0						
Uruwa: Minimal	−0.10	−0.20	−0.00	20.23	0.95	0.17
Uruwa: Lutheran	−0.09	−0.17	−0.02	59.61	0.98	0.13
Uruwa: SDA	−0.09	−0.15	−0.04	316.46	1.00	0.07
Sydney: Non-mus	−0.28	−0.36	−0.21	>19999.00	1.00	0.00
Sydney: Musician	−0.86	−1.06	−0.66	>19999.00	1.00	0.00
Effect of Δ*harmonicity* > 0						
Uruwa: Minimal	−0.06	−0.16	0.05	0.22	0.18	0.38
Uruwa: Lutheran	−0.08	−0.16	0.00	0.05	0.05	0.22
Uruwa: SDA	−0.00	−0.07	0.07	1.11	0.53	0.71
Sydney: Non-mus	0.05	−0.02	0.12	8.55	0.90	0.44
Sydney: Musician	0.15	0.00	0.30	21.65	0.96	0.09
Effect of Δ*mean pitch* > 0						
Uruwa: Minimal	−0.08	−0.26	0.10	0.29	0.22	0.24
Uruwa: Lutheran	0.11	−0.02	0.24	10.55	0.91	0.18
Uruwa: SDA	−0.03	−0.13	0.07	0.41	0.29	0.48
Sydney: Non-mus	−0.15	−0.25	−0.04	0.01	0.01	0.05
Sydney: Musician	−0.03	−0.32	0.27	0.76	0.43	0.20

Results from a frequentist model

For those unfamiliar with Bayesian modelling, in Table A6, we provide a frequentist version of the explanatory Bayesian model reported in the main text. It is slightly simplified in that it has only a random intercept by participant (as is often the case with frequentist models, convergence fails when random slopes are also included). A separate model was fitted to each of the five participant groups – in glmer, this is necessary because there is no syntax to ensure random effects are estimated only within and not between exposure groups (when exposure group is entered as an interaction, as in the original model).

Table A6. Summary of the frequentist version of the main explanatory model. As in the original Bayesian model, the predictor trial_type (which codes for dyads versus triads) is sum-coded, hence the results for diff_roughness, diff_harmonicity, and diff_mean_pitch are main effects over both dyads and triads. Roughness (and only roughness) is significant in every exposure group.

		**Estimate**	**Std. Error**	**z value**	**Pr(> |z|)**
Uruwa: Minimal	(Intercept)	−0.10	0.27	−0.36	0.718
	diff_roughness	−0.12*	0.06	−2.23	0.026
	diff_harmonicity	−0.05	0.06	−0.89	0.373
	diff_mean_pitch	−0.11	0.10	−1.13	0.257
	trial_typeTriads	−0.04	0.05	−0.79	0.428
	diff_roughness:trial_typeTriads	−0.03	0.06	−0.47	0.635
	diff_harmonicity:trial_typeTriads	−0.08	0.06	−1.39	0.165
	diff_mean_pitch:trial_typeTriads	−0.02	0.10	−0.17	0.867
	SD (Intercept participant)	1.24			
Uruwa: Lutheran	(Intercept)	0.24	0.16	1.49	0.137
	diff_roughness	−0.08*	0.04	−2.16	0.031
	diff_harmonicity	−0.08+	0.04	−1.80	0.072
	diff_mean_pitch	0.12+	0.07	1.77	0.076
	trial_typeTriads	−0.05	0.04	−1.43	0.154
	diff_roughness:trial_typeTriads	0.02	0.04	0.52	0.604
	diff_harmonicity:trial_typeTriads	0.01	0.04	0.30	0.767
	diff_mean_pitch:trial_typeTriads	0.05	0.07	0.72	0.471
	SD (Intercept participant)	0.98			
Uruwa: SDA	(Intercept)	0.37*	0.17	2.11	0.035
	diff_roughness	−0.10**	0.03	−3.26	0.001
	diff_harmonicity	−0.01	0.03	−0.17	0.863
	diff_mean_pitch	−0.03	0.05	−0.65	0.518
	trial_typeTriads	−0.06*	0.03	−1.97	0.049
	diff_roughness:trial_typeTriads	0.08**	0.03	2.82	0.005
	diff_harmonicity:trial_typeTriads	0.07*	0.03	2.24	0.025
	diff_mean_pitch:trial_typeTriads	0.05	0.05	0.99	0.322
	SD (Intercept participant)	1.53			
Sydney: Non-mus	(Intercept)	0.29***	0.07	4.31	<0.001
	diff_roughness	−0.27***	0.03	−8.91	<0.001
	diff_harmonicity	0.05	0.03	1.48	0.139
	diff_mean_pitch	−0.15**	0.05	−2.86	0.004
	trial_typeTriads	−0.07*	0.03	−2.45	0.014
	diff_roughness:trial_typeTriads	−0.05	0.03	−1.58	0.114
	diff_harmonicity:trial_typeTriads	−0.01	0.03	−0.25	0.800
	diff_mean_pitch:trial_typeTriads	0.03	0.05	0.65	0.519
	SD (Intercept participant)	0.47			
Sydney: Musician	(Intercept)	0.14*	0.07	1.98	0.048
	diff_roughness	−0.75***	0.06	−12.67	<0.001
	diff_harmonicity	0.14*	0.06	2.42	0.015
	diff_mean_pitch	−0.04	0.10	−0.40	0.690
	trial_typeTriads	−0.16**	0.05	−3.28	0.001
	diff_roughness:trial_typeTriads	0.10+	0.06	1.74	0.082
	diff_harmonicity:trial_typeTriads	−0.01	0.06	−0.17	0.866
	diff_mean_pitch:trial_typeTriads	0.26**	0.10	2.74	0.006
	SD (Intercept participant)	0.20			

+ $p < 0.1$, * $p < 0.05$, ** $p < 0.01$, *** $p < 0.001$

Additional plots

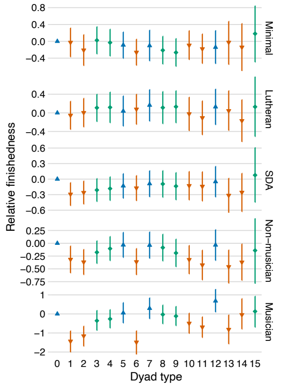

(a) All tested dyads disregarding timbre and transposition.

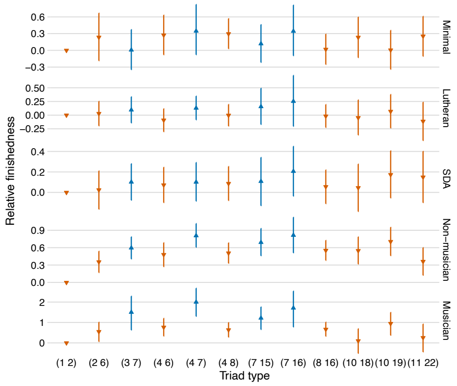

(b) All tested triads disregarding timbre and transposition.

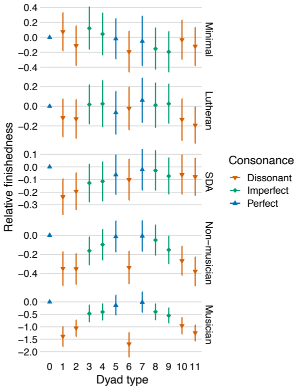

(c) All tested dyads disregarding timbre, transposition, and the upper pitch's octave.

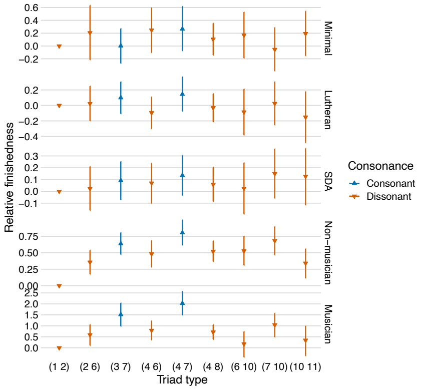

(d) All tested triads disregarding timbre, transposition, and the upper pitches' octaves.

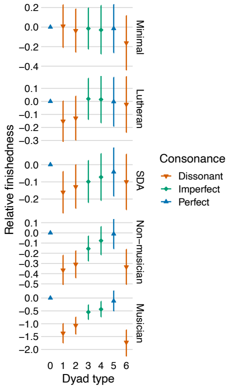

(e) All tested dyads disregarding timbre, transposition, and all pitches' octaves; i.e., T_n-type.

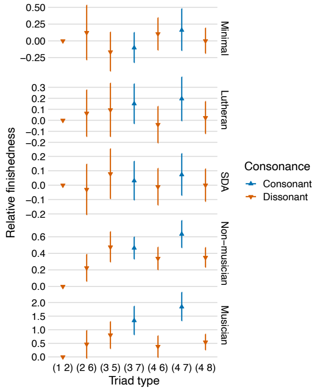

(f) All tested triads disregarding timbre, transposition, and all pitches' octaves; i.e., T_n-type.

Fig A2. The relative stabilities of individual chords, at different levels of abstraction, as estimated with a multilevel Bayesian Thurstone model. This is the same as Fig A3, but with the plots' vertical scales varying between exposure groups to facilitate comparisons of within-group effects. See Fig 3 in the main text for further information.

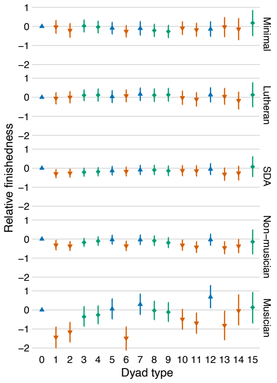

(a) All tested dyads disregarding timbre and transposition.

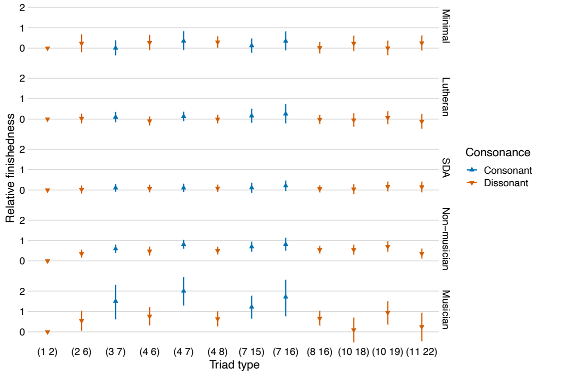

(b) All tested triads disregarding timbre and transposition.

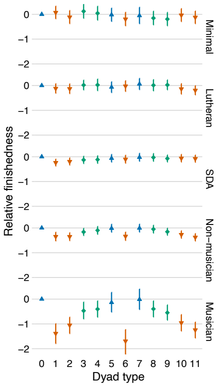

(c) All tested dyads disregarding timbre, transposition, and the upper pitch's octave.

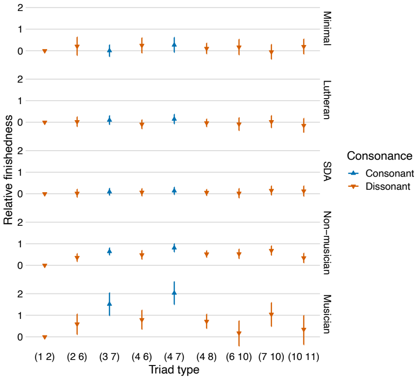

(d) All tested triads disregarding timbre, transposition, and the upper pitches' octaves.

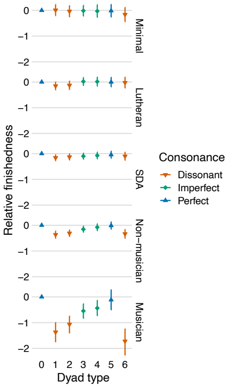

(e) All tested dyads disregarding timbre, transposition, and all pitches' octaves; i.e., T_n-type.

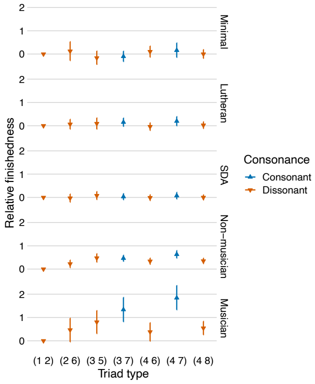

(f) All tested triads disregarding timbre, transposition, and all pitches' octaves; i.e., T_n-type.

Fig A3. The relative stabilities of individual chords, at different levels of abstraction, as estimated with a multilevel Bayesian Thurstone model. This the same as Fig A2, but with the plots' vertical scales kept identical between exposure groups to facilitate comparisons of between-group effects. See Fig 3 in the main text for further information.

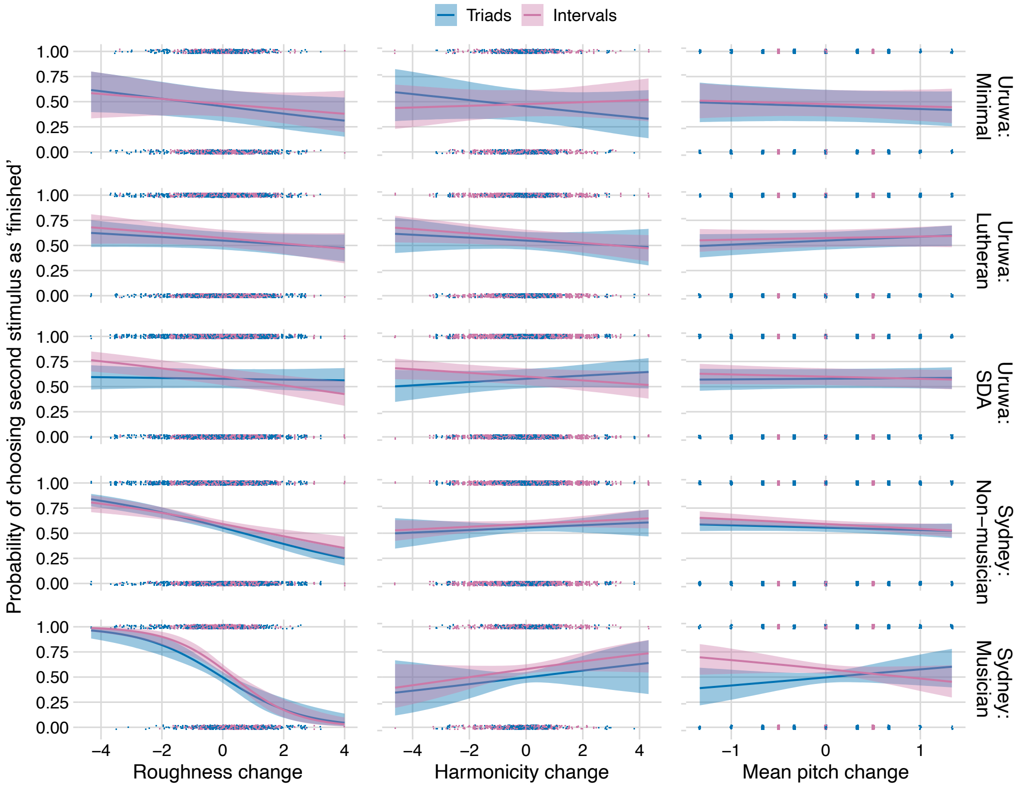

Fig A4. For dyads and triads, the effect of changes in roughness, harmonicity, and mean pitch on the probability of choosing the stimulus as finished. The same as Fig 4 in the main text, except showing dyads and triads separately and using ribbons to show the 95% Bayesian credibility intervals.

References

1. Carey N, Clampitt D. Aspects of well-formed scales. Music Theory Spectrum. 1989;11(2):187–206. doi:10.1525/mts.1989.11.2.02a00030.

2. Kaeppler AL, Stillman AK, Tsounis D, Falk C, O'Donnell D, Barwick L, et al. Introduction to Oceania and its Music: Musical Migrations. In: Kaeppler AL, Love JW, editors. The Garland Encyclopedia of World Music: Australia and the Pacific Islands. vol. 9. Garland Publishing Inc.; 1998. p. 54–69.

3. Sarvasy HS. A Grammar of Nungon: A Papuan Language of Northeast New Guinea. Brill; 2017.

4. Kaeppler AL, Niles D. The music and dance of New Guinea. In: Kaeppler AL, Love JW, editors. The Garland Encyclopedia of World Music: Australia and the Pacific Islands. vol. 9. Garland Publishing Inc.; 1998. p. 498–513.

5. Crowdy D. Guitar Style, Open Tunings, and Stringband Music in Papua New Guinea. Apwitihire: Studies in Papua New Guinea musics. Boroko, PNG: Institute of Papua New Guinea Studies; 2005.

6. Webb M. *Palang* conformity and *fulset* freedom: Encountering Pentecostalism's "sensational" liturgical forms in the postmissionary church in Lae, Papua New Guinea. Ethnomusicology. 2011;55(3):445–472.

7. Mulak KE, Sarvasy HS, Tuninetti A, Escudero P. Word learning in the field: Adapting a laboratory-based task for testing in remote Papua New Guinea. PLOS One. 2021;16(9):1–25. doi:10.1371/journal.pone.0257393.

8. Sarvasy HS, Morgan AM, Yu J, Ferreira VS, Momma S. Cross-clause chaining in Nungon, Papua New Guinea: Evidence from eye-tracking. Memory and Cognition. 2022;doi:10.3758/s13421-021-01253-3.

9. Lartillot O, Toiviainen P, Eerola T. A Matlab Toolbox for Music Information Retrieval. In: Preisach C, Burkhardt H, Schmidt-Thieme L, Decker R, editors. Data Analysis, Machine Learning and Applications. Springer-Verlag; 2008. p. 261–268.

10. Sethares WA. Tuning, Timbre, Spectrum, Scale. 2nd ed. London: Springer Verlag; 2005.

11. Plomp R, Levelt WJM. Tonal consonance and critical bandwidth. The Journal of the Acoustical Society of America. 1965;38(4):548–560. doi:10.1121/1.1909741.

12. Milne AJ, Laney R, Sharp DB. Testing a spectral model of tonal affinity with microtonal melodies and inharmonic spectra. Musicae Scientiae. 2016;20(4):465–494. doi:10.1177/1029864915622682.

13. Harrison PMC, Pearce MT. Simultaneous consonance in music perception and composition. Psychological Review. 2020;127(2):216–244. doi:10.1037/rev0000169.

14. Milne AJ, Bulger D, Herff SA. Exploring the space of perfectly balanced rhythms and scales. Journal of Mathematics and Music. 2017;11(2–3):101–133. doi:10.1080/17459737.2017.1395915.

15. Smit EA, Milne AJ, Dean RT, Weidemann G. Perception of affect in unfamiliar musical chords. PLOS One. 2019;14(6):1–28. doi:10.1371/journal.pone.0218570.

16. R Core Team. R: A language and environment for statistical computing; 2014. Available from: `http://www.R-project.org/`.

17. Bürkner PC. brms: An R Package for Bayesian Multilevel Models Using Stan. Journal of Statistical Software. 2017;80(1):1–28. doi:10.18637/jss.v080.i01.

18. Bürkner PC. Advanced Bayesian Multilevel Modeling with the R Package brms. The R Journal. 2018;10(1):395–411.

19. Carpenter B, Gelman A, Hoffman MD, Lee D, Goodrich B, Betancourt M, et al. Stan: A probabilistic programming language. Journal of Statistical Software. 2017;76(1):10.18637/jss.v076.i01.

20. Critchlow DE, Fligner MA. Paired comparison, triple comparison, and ranking experiments as generalized linear models, and their implementation on GLIM. Psychometrika. 1991;56(3):517–533.

21. Marsman M, Wagenmakers EJ. Three insights from a Bayesian interpretation of the one-sided P value. Educational and Psychological Measurement. 2017;77(3):529–539. doi:10.1177/0013164416669201.

22. Makowski D, Ben-Shachar MS, Chen SHA, Lüdecke D. Indices of effect existence and significance in the Bayesian framework. Frontiers in Psychology. 2019;10:2767. doi:10.3389/fpsyg.2019.02767.

23. Vehtari A, Gelman A, Gabry J. Practical Bayesian model evaluation using leave-one-out cross-validation and WAIC. Statistics and Computing. 2017;27(5):1413–1432.

24. Kruschke JK. Doing Bayesian Data Analysis: A Tutorial with R, JAGS, and Stan. 2nd ed. London, UK: Academic Press; 2014.
