# Supplementary material for: Evidence for a universal association of auditory roughness with musical stability
Source: PLoS One. 2023 Sep 20;18(9):e0291642. doi: 10.1371/journal.pone.0291642 (PMC10511120; doi:10.1371/journal.pone.0291642)
Supplement: Attachment — Submitted filename: Response_to_reviewers.pdf [file pone.0291642.s002.pdf]

Dear Dr. Grahn,

Thank you for the positive comments and the invitation to revise and resubmit. We also thank the reviewers for genuinely constructive criticisms and suggestions, which have improved the manuscript. Below, I have copied the original letter with our responses to each point added in blue. The formatting of the revised manuscripts (the clean version and the changes-marked version) has been changed as requested in point 1 in the letter below.

Yours faithfully,

Andrew Milne

PONE-D-23-12398
Evidence for a universal association of auditory roughness with musical dissonance
PLOS ONE

Dear Dr. Milne,

Thank you for submitting your manuscript to PLOS ONE. After careful consideration, we feel that it has merit but does not fully meet PLOS ONE's publication criteria as it currently stands. Therefore, we invite you to submit a revised version of the manuscript that addresses the points raised during the review process.

As you can see, the reviewers both felt very positively about the manuscript, as did I. Most of the suggestions are to add clarity and consider some definitional distinctions that are made, comparing to other types of distinctions made in the literature, which will hopefully expand the audience for this paper.

Please submit your revised manuscript by Aug 10 2023 11:59PM. If you will need more time than this to complete your revisions, please reply to this message or contact the journal office at plosone@plos.org. When you're ready to submit your revision, log on to https://www.editorialmanager.com/pone/ and select the 'Submissions Needing Revision' folder to locate your manuscript file.

Please include the following items when submitting your revised manuscript:

- A rebuttal letter that responds to each point raised by the academic editor and reviewer(s). You should upload this letter as a separate file labeled 'Response to Reviewers'.
- A marked-up copy of your manuscript that highlights changes made to the original version. You should upload this as a separate file labeled 'Revised Manuscript with Track Changes'.
- An unmarked version of your revised paper without tracked changes. You should upload this as a separate file labeled 'Manuscript'.

If you would like to make changes to your financial disclosure, please include your updated statement in your cover letter. Guidelines for resubmitting your figure files are available below the reviewer comments at the end of this letter.

If applicable, we recommend that you deposit your laboratory protocols in protocols.io to enhance the reproducibility of your results. Protocols.io assigns your protocol its own identifier (DOI) so that it can be cited independently in the future. For instructions see: https://journals.plos.org/plosone/s/submission-guidelines#loc-laboratory-protocols.

Additionally, PLOS ONE offers an option for publishing peer-reviewed Lab Protocol articles, which describe protocols hosted on protocols.io. Read more information on sharing protocols at https://plos.org/protocols?utm_medium=editorial-email&utm_source=authorletters&utm_campaign=protocols.

We look forward to receiving your revised manuscript.

Kind regards,

Jessica Adrienne Grahn
Academic Editor
PLOS ONE

Journal requirements:

When submitting your revision, we need you to address these additional requirements.

1. Please ensure that your manuscript meets PLOS ONE's style requirements, including those for file naming. The PLOS ONE style templates can be found at

https://journals.plos.org/plosone/s/file?id=wjVg/PLOSOne_formatting_sample_main_body.pdf and

https://journals.plos.org/plosone/s/file?id=ba62/PLOSOne_formatting_sample_title_authors_affiliations.pdf

The formatting has been updated.

2. lease include a complete copy of PLOS' questionnaire on inclusivity in global research in your revised manuscript. Our policy for research in this area aims to improve transparency in the reporting of research performed outside of researchers' own country or community. The policy applies to researchers who have travelled to a different country to conduct research, research with Indigenous populations or their lands, and research on cultural artefacts. The questionnaire can also be requested at the journal's discretion for any other submissions, even if these conditions are not met. Please find more information on the policy and a link to download a blank copy of the questionnaire here: https://journals.plos.org/plosone/s/best-practices-in-research-reporting. Please upload a completed version of your questionnaire as Supporting Information when you resubmit your manuscript.

This has been uploaded as part of the online submission.

3. We note that Figure 2 in your submission contain copyrighted images. All PLOS content is published under the Creative Commons Attribution License (CC BY 4.0), which means that the manuscript, images, and Supporting Information files will be freely available online, and any third party is permitted to access, download, copy, distribute, and use these materials in any way, even commercially, with proper attribution. For more information, see our copyright guidelines: http://journals.plos.org/plosone/s/licenses-and-copyright.

We require you to either (1) present written permission from the copyright holder to publish these figures specifically under the CC BY 4.0 license, or (2) remove the figures from your submission:

a. You may seek permission from the original copyright holder of Figure 2 to publish the content specifically under the CC BY 4.0 license.

We recommend that you contact the original copyright holder with the Content Permission Form (http://journals.plos.org/plosone/s/file?id=7c09/content-permission-form.pdf) and the following text:

“I request permission for the open-access journal PLOS ONE to publish XXX under the Creative Commons Attribution License (CCAL) CC BY 4.0 (http://creativecommons.org/licenses/by/4.0/). Please be aware that this license allows unrestricted use and distribution, even commercially, by third parties. Please reply and provide explicit written permission to publish XXX under a CC BY license and complete the attached form.”

Please upload the completed Content Permission Form or other proof of granted permissions as an "Other" file with your submission.

In the figure caption of the copyrighted figure, please include the following text: “Reprinted from [ref] under a CC BY license, with permission from [name of publisher], original copyright [original copyright year].”

b.If you are unable to obtain permission from the original copyright holder to publish these figures under the CC BY 4.0 license or if the copyright holder’s requirements are incompatible with the CC BY 4.0 license, please either i) remove the figure or ii) supply a replacement figure that complies with the CC BY 4.0 license. Please check copyright information on all replacement figures and update the figure caption with source information. If applicable, please specify in the figure caption text when a figure is similar but not identical to the original image and is therefore for illustrative purposes only.

This is now Fig 3. The photograph was taken by my daughter Astra Milne (currently age 17) and she has provided permission for it to published under a CC B Y 4.0 license.

4. We note that Figure 1 in your submission contain [map/satellite] images which may be copyrighted. All PLOS content is published under the Creative Commons Attribution License (CC BY 4.0), which means that the manuscript, images, and Supporting Information files will be freely available online, and any third party is permitted to access, download, copy, distribute, and use these materials in any way, even commercially, with proper attribution. For these reasons, we cannot publish previously copyrighted maps or satellite images created using proprietary data, such as Google software (Google Maps, Street View, and Earth). For more information, see our copyright guidelines: http://journals.plos.org/plosone/s/licenses-and-copyright.

We require you to either (1) present written permission from the copyright holder to publish these figures specifically under the CC BY 4.0 license, or (2) remove the figures from your submission:

a. You may seek permission from the original copyright holder of Figure 1 to publish the content specifically under the CC BY 4.0 license.

We recommend that you contact the original copyright holder with the Content Permission Form (http://journals.plos.org/plosone/s/file?id=7c09/content-permission-form.pdf) and the following text:

“I request permission for the open-access journal PLOS ONE to publish XXX under the Creative Commons Attribution License (CCAL) CC BY 4.0 (http://creativecommons.org/licenses/by/4.0/). Please be aware that this license allows unrestricted use and distribution, even commercially, by

third parties. Please reply and provide explicit written permission to publish XXX under a CC BY license and complete the attached form.”

Please upload the completed Content Permission Form or other proof of granted permissions as an "Other" file with your submission.

In the figure caption of the copyrighted figure, please include the following text: “Reprinted from [ref] under a CC BY license, with permission from [name of publisher], original copyright [original copyright year].”

b. If you are unable to obtain permission from the original copyright holder to publish these figures under the CC BY 4.0 license or if the copyright holder’s requirements are incompatible with the CC BY 4.0 license, please either i) remove the figure or ii) supply a replacement figure that complies with the CC BY 4.0 license. Please check copyright information on all replacement figures and update the figure caption with source information. If applicable, please specify in the figure caption text when a figure is similar but not identical to the original image and is therefore for illustrative purposes only.

The following resources for replacing copyrighted map figures may be helpful:

USGS National Map Viewer (public domain): http://viewer.nationalmap.gov/viewer/

The Gateway to Astronaut Photography of Earth (public domain): http://eol.jsc.nasa.gov/sseop/clickmap/

Maps at the CIA (public domain): https://www.cia.gov/library/publications/the-world-factbook/index.html and https://www.cia.gov/library/publications/cia-maps-publications/index.html

NASA Earth Observatory (public domain): http://earthobservatory.nasa.gov/

Landsat: http://landsat.visibleearth.nasa.gov/

USGS EROS (Earth Resources Observatory and Science (EROS) Center) (public domain): http://eros.usgs.gov/#

Natural Earth (public domain): http://www.naturalearthdata.com/

This is now Fig 2. The map is hand-drawn by author Hannah Sarvasy and does not contain any copyrighted images.

5. Please review your reference list to ensure that it is complete and correct. If you have cited papers that have been retracted, please include the rationale for doing so in the manuscript text, or remove these references and replace them with relevant current references. Any changes to the reference list should be mentioned in the rebuttal letter that accompanies your revised manuscript. If you need to cite a retracted article, indicate the article’s retracted status in the References list and also include a citation and full reference for the retraction notice.

The reference list is correct and complete. Any new citations are mentioned in the letter below or are marked blue in the revised manuscript.

[Note: HTML markup is below. Please do not edit.]

Reviewers' comments:

Reviewer's Responses to Questions

Comments to the Author

1. Is the manuscript technically sound, and do the data support the conclusions?

The manuscript must describe a technically sound piece of scientific research with data that supports the conclusions. Experiments must have been conducted rigorously, with appropriate controls, replication, and sample sizes. The conclusions must be drawn appropriately based on the data presented.

Reviewer #1: Yes

Reviewer #2: Partly

---

2. Has the statistical analysis been performed appropriately and rigorously?

Reviewer #1: Yes

Reviewer #2: Yes

---

3. Have the authors made all data underlying the findings in their manuscript fully available?

The PLOS Data policy requires authors to make all data underlying the findings described in their manuscript fully available without restriction, with rare exception (please refer to the Data Availability Statement in the manuscript PDF file). The data should be provided as part of the manuscript or its supporting information, or deposited to a public repository. For example, in addition to summary statistics, the data points behind means, medians and variance measures should be available. If there are restrictions on publicly sharing data—e.g. participant privacy or use of data from a third party—those must be specified.

Reviewer #1: Yes

Reviewer #2: Yes

---

4. Is the manuscript presented in an intelligible fashion and written in standard English?

PLOS ONE does not copyedit accepted manuscripts, so the language in submitted articles must be clear, correct, and unambiguous. Any typographical or grammatical errors should be corrected at revision, so please note any specific errors here.

Reviewer #1: Yes

Reviewer #2: Yes

5. Review Comments to the Author

Please use the space provided to explain your answers to the questions above. You may also include additional comments for the author, including concerns about dual publication, research ethics, or publication ethics. (Please upload your review as an attachment if it exceeds 20,000 characters)

Reviewer #1: Dear authors, I read with very much interest your manuscript on how roughness is perceived across individuals with different exposure to Western sonorities. The research design is clear and well presented. The findings are relevant for the literature on the cross-cultural investigation of consonance and dissonance perception. There is only one point that I would like you to think about in the revision of the paper, which relates with the way in which you seemingly conceive the dichotomy between culture and nature. You will find more details in the following list of suggestions, together with a couple of recent important references that are missing.

Our fundamental distinction is universal–cultural (not nature–culture) but our use of the word "natural" had obfuscated that. That word is no longer used. In the Introduction, we have added a new paragraph and figure to provide a formal definition of how we use those terms and relate them to prior literature. The reason we prefer universal–cultural over nature/biology/innate–culture is for precisely the point you correctly make later, which is that a universal response can be learned from cultural exposure (so long as that cultural exposure is itself essentially universal). Also, the new definition makes clear that these two aspects are not opposites and can run in parallel. We had not been clear about these before, but hopefully these are now more clear in the following text (there is also a new Figure 1 in the main text):

"To be more formal about our definitions of 'cultural', 'non-cultural', and 'universal' effects, let us label a participant's affective response as Y (e.g., pleasantness or stability), a psychoacoustic feature as X (e.g., roughness or harmonicity), the participant's culture as C, and a cultural mechanism as M (e.g., the participant's long-term familiarity with that value of X or their learned associations with that level of X, given their culture C). For a given stimulus, the value of the psychoacoustic feature X does not differ between cultures (it may differ slightly between individuals with normal hearing but not in aggregate in a given culture) whereas, by definition, a feature's culture-dependent distribution M may differ (e.g., in Western music, harmonic timbres are more common than inharmonic; in gamelan music, inharmonic timbres are more common). Causally, M mediates the effect of X; as shown in Figure 1. The direct path from X to Y would be a non-cultural effect of X, the mediated path from M to Y would be a cultural effect of X. For any psychoacoustic feature X, both types of effect – non- cultural and cultural – can run in parallel (and may have different strengths); putative mechanisms were exemplified in the previous two paragraphs. The non-cultural effect would be universal (this follows from its definition), but a cultural effect could also be universal if the associated feature's distribution does not change between cultures; for example, human speech is a cultural universal and has high harmonicity, which may encourage a universal preference for chords with high harmonicity (Bowling et al., 2018). However, there would be no ethical way of experimentally changing long-term exposure to speech, or another cultural universal, so as to uncover its precise effect on music-induced affect. We prefer the universal–cultural framing, also used in Fritz et al. (2009); Egermann et al. (2015); Jacoby et al. (2019); McPherson et al. (2020); Athanasopoulos et al. (2021), over the biology/innate–culture framing in McDermott and Hauser (2005); Sievers et al. (2013); Milne (2013); McDermott et al. (2016); Bowling et al. (2017, 2018) because the latter may not be amenable to experimental testing."

1) The recent review on C/D by Di Stefano, Vuust, and Brattico (2022, Physics of Life Reviews) is relevant both as general reference for the introduction and, more importantly, for the discussion.

Added.

2) Is fusion also worth mentioning as a proxy for consonance in the introduction?

Added on p.1.

3) Please consider adding “harmonic” before “intervals” when introducing dyads on page 2 second paragraph. Again, regarding the putative mechanisms underlying C/D please refer to Di Stefano et al., 2022 (and probably expand the paragraph a bit).

Both added.

4) “A listener may feel a major chord is more pleasant simply because they have heard that chord more often in their culture’s music”: please support this claim, as the link between exposure and pleasantness is not self-evident (e.g., Zajonc, R. B. (1968). Attitudinal effects of mere exposure. Journal of personality and social psychology, 9, 1.).

We have added citations to Zajonc and Parncutt to provide more context and support to the claim: “An alternative explanation is that these differing consonances and dissonances are driven by cultural exposure – via familiarity (mere exposure, Zajonc 1968) and associative learning. For example, a listener may feel a major chord is more pleasant simply because they have heard that chord more often in their culture’s music (Parncutt, 2014) …”

5) As observed in the general comment above, it is assumed that there is an obvious alternative between cultural vs universal origins of C/D. I know that this is often the way how the issue is presented in the debate. However, I don’t think these two terms have to be taken as opposites. Something cultural can be well universal, if we mean with universal “universally widespread across cultures and individuals”. Aren’t music and artistic practices themselves universal and cultural at the same time?

We agree and hadn’t intended these to be seen as opposites. Hopefully this is now made clear by the new paragraph and figure in the Introduction.

When reporting on the study by McDermott et al. (2016), you wrote that it seems to suggest a purely cultural mechanism for C/D. In similar occurrences, it is not entirely clear what “purely cultural” means. Moreover, what “for C/D” refers to in the previous sentence? Discrimination? Perception? Preference? Use? Please also mention the papers that raised issues regarding the nature paper (e.g., Bowling, D. L., Hoeschele, M., Gill, K. Z., & Fitch, W. T. (2017). The nature and nurture of musical consonance. Music Perception: An Interdisciplinary Journal, 35(1), 118-121.)

The intended meaning of “purely cultural effect” should now be clear from the preceding text (quoted above), and the text has been changed to: “The second is more plausible: a recent investigation with the Tsimanè – a remote Bolivian community with minimal exposure to Western music – found they did not rate intervals and chords deemed consonant in Western music theory as more pleasant (McDermott et al., 2016). This suggests perceptions of C/D may be a purely cultural effect. However, in that study, ratings were requested for the ‘pleasantness’ of different dyads and triads, not their ‘stability’ (or some other related term such as ‘closure’, ‘tension’, or ‘finishedness’). (Bowling et al. (2017) also raise several possible methodological issues.)”

6) “In our studies, we typically use a smaller group of musicians compared to non-musicians, as their responses are generally very consistent and even small effects are detectable in a cohort of this size” (p. 5). I agree but probably you need to support the claim.

This has been slightly reworded and a citiation added: “In our studies, we typically use a smaller group of musicians compared to non-musicians, as their responses are generally more consistent (Lahdelma and Eerola, 2020b) so even small effects are detectable in a cohort of this size.”

7) “Of those, only one participant (the Arabic music listener) did not indicate listening to any Western music genre.” Not sure I understand the meaning of this sentence.

This has been revised: “All participants except one (the Arabic music listener) listen to at least one Western music genre”

8) P. 9: “A total of 27% of blocks were removed for the Uruwa participants.” It seems a significant portion, can you provide some comments on this?

We checked whether the removal of the data impacted the reported results by fitting an exploratory model to all the data (without any blocks removed). This model has very similar posterior means and every strongly evidenced hypothesis remains strongly evidenced. This model and the outcome are summarized in the Appendix.

9) Discussion, line 2: I would avoid the use of the (umbrella) term ‘natural’ here. How about ‘consistent’?

Every instance of “natural” has been replaced by “non-arbitrary” or, when the phrasing is reversed, “arbitrary”, or “reasonable”.

10) When discussing roughness, you might consider the recent review by Di Stefano, N., & Spence, C. (2022). Roughness perception: A multisensory/crossmodal perspective. Attention, Perception, & Psychophysics, 84(7), 2087-2114.

Added.

11) “the association between harmonicity and stability is not a natural one but is learned”, p. 13. Again, I would recommend rephrasing similar claims, as they seem conceptually problematic. What we learn can be natural (e.g., language, walking…). At the same time, universal traits are not necessarily natural, can be learned… In general, in the discussion, I would tone a bit down the emphasis on the cultural vs natural or natural vs acquired framework… Again, we learn to do many things that are considered as natural.

We no longer use the term “natural” in these contexts and hopefully these points are now addressed by the new material in the Introduction.

12) “The presence of a distinct effect of roughness in even the most musically remote PNG community suggests a universal, or at least non-arbitrary, association between roughness and instability.” P. 14. How about a “consistent perceptual association between roughness and instability”? or something like? This is changed.
Another reason why I don’t like the cultural/learned perspective. Consider the case of a cubist female portrait. We need to learn how to see it, how to associate those strange geometrical figures to a nose here, an eye there, an asymmetric and misplaced breast. After “learning”, the portrait will become clearer. However, we will only have learnt how to decipher it, but the portrait will still seem an uncommon, weird way of representing a female body. Something similar might happen with music, with cultural learning that might facilitate the understanding of serialism or alea, but will not make it sound like perfect consonances...

Hopefully the clarified definitions in the Intro deal with this issue. But in this example, there is surely an argument that there are universal aspects – the relationship between the subject and

its representation are more obscure than in a realistic portrait and hence there is probably greater cognitive load and complexity. But the extent to which this additional complexity is deemed pleasurable, for example, is both individual and also influenced the viewer's cultural expectations and familiarity with cubist art.

Reviewer #2: The paper is really well written, the topic is relevant, and the experiments and statistical analyses are sound. My only serious concern is the proxy between dissonance and "sensation of closure".

Major
In my view the question of whether c/d is universal or culture-dependent can be considered as two problems: 1) whether the perception of c/d is universal; and 2) whether the associations of X with c/d are universal. I think most intercultural studies in the past have tackle the second point, and specifically whether pleasantness is universally associated to consonance. In my view, the current paper studies whether the feeling of cloure is universally associated to dissonance (Figure 3 suggests it is not); and whether certain markers that have been related to dissonance in the past (roughness, harmonicity, ...) are universatlly associated to this feeling of closure (Table 1 suggests roughness is). I think these results are relevant and, to my eyes, they look quite robust. On the other hand, I disagree that this can be interpreted as "evidence for a universal association of auditory roughness with musical dissonance". This conclusion depends on the implicit assumption that dissonance = "feeling of closure"; however, the demonstration that the feeling of closure is not universally associated with dissonance of Fig. 3 seems to contradict this implicit assumption.

Another potential problem is the implicit association of "stability" with "feeling of closure". I think the problem here is that "stability" can be interpreted in two ways: 1) whether the sound is static; 2) whether the sound can be represented stable point (in the sense of dynamic systems). I can see why in the second interpretation a stable sound is intrinsically more likely to feel like a finishing sound than an unstable sound; however, I think this is not necessarily the case in the first interpretation (even if this happen to be the case in Western music). I have the impression that the association of "unstability" with "dissonance" stems rather from the beating sensation of the dissonant chords, which would correspond to the first interpretation.

In my view, the report would be more rigorous avoiding these implicit associations: namely, just stating that "closure/finishing sensation" rather than "dissonance" or "stability" is associated to roughness; and removing the implicit associations between one and the other in the MS. Critically, this would affect not only the abstract, introduction, and discussion, but most importantly also the title.

We changed the title and multiple instances where we solely referred to stability in the abstract, introduction and discussion. Much of the introduction is still framed in terms of consonance/dissonance and stability but we make clear that our operationalization is "finishedness", and we use focus on that term when reporting the Results.

Minor
I have the impression the paper is very verbose explaining the Valley, the travels, and the presentation procedure, but pushed all the technical methods to the appendix. I would suggest to ensure that the methods section contains at least all the information necessary to reproduce the analyses. I think pushing part of the procedures and the cultural description to the appendix (more generally, shortening the paper) will increase the chances that hardcore psychophysics researchers (which I reckon will be the most interested in the results) read the paper, but of course that is up to the authors to decide.

Thank you for the suggestion and we understand the reviewer's perspective. However, we decided to keep the current structure as the cross-cultural context in which the research was conducted is extremely important for all interested readers to know before reading the results, whereas the methods and the technical details can be easily looked up by those who are interested.

I am not familiar with Bayesian multilevel logistic regression, so the following questions should be understood as "more details are needed for unfamiliar readers to fully understand the methodology" is they are misguided:
1) Is the evidence ratio really the quotient between the posteriors? To my understanding the evidence is p(data|hypothesis), whereas the posterior is p(hypothesis|data). If the priors for all hypotheses are the same, the ratio between two evidences equals the ratio between two posteriors, but the definition is the former, no? Is not the ratio of the evidences simply Bayes' K factor? As far as I understand K factors are the standard way to report Bayesian results, wouldn't it be better to adhere to this standard notation?

We think there are two understandable misunderstandings here. The term "evidence ratio" comes from the brms package (the most-used Bayesian modelling package in R, and what we use), which uses this term for the ratio between the posterior probability an effect is positive (> 0) versus negative (< 0) (or vice versa). However, we realise the term "evidence" is sometimes used to denote the denominator (aka, marginal likelihood) in the Bayes formula. But that's not what is being referred to here. Also, this brms evidence ratio is not a standard Bayes factor, which is the ratio of evidence between a point null and a non-null. Along with many Bayesians in the Gelman and Kruschke "tradition", we are wary of traditional Bayes factors for three reasons – they are hard to calculate accurately, it is implausible a point null is ever exactly true in behavioural or sociological experiments, they are very sensitive to the priors chosen. This is why we use Kruschke's ROPE to quantify evidence for a range of values practically equivalent to 0.

To clarify these and other things, we have expanded our p.10 description of the stats related stuff:

"We use *posterior probabilities* and *evidence ratios* ('Post.p' and 'Evid.Ratio' in Table 1) to assess evidence in favour of directional hypothesis tests. For example, given a hypothesis that roughness reduces finishedness, we use the posterior probability that the effect of roughness is less than zero to quantify our certainty that this hypothesis is correct. Let us imagine that posterior probability is 0.89; this would mean that (given the model) there is a probability of 0.89 that roughness has a negative effect, and a probability of 1 – 0.89 = 0.11 it has a positive effect. This can also be quantified as an evidence ratio, which are the odds in favour of the hypothesis so, for this imaginary example, the evidence ratio that roughness has a negative effect would be 0.89/0.11 = 8.09. For a directional hypothesis like this, an evidence ratio > 19 (a posterior probability > 0.95) is somewhat analogous to a p-value < 0.05 (Marsman and Wagenmakers, 2017; Makowski et al., 2019), and we refer to such a ratio as 'strong evidence'; for a bidirectional exploratory hypothesis, this threshold is > 39 (a posterior probability of 0.975 for one direction and 0.025 for the opposite direction, hence the total probability is still 0.95).

"We do not use Bayes factors to test evidence for and against a point-null hypothesis, instead preferring the *region of practical equivalence* (ROPE) approach recommended by Kruschke (2014), which is to choose a range of effect sizes that are so small, they can be considered practically equivalent to zero. An advantage of this approach is that it is substantially less sensitive to the prior specification than are Bayes factors (Kruschke, 2014); it also allows domain expertise to be used to determine a plausible region of practical equivalence. For our predictors, we define the ROPE as being in the interval [0.036, 0.036] (the standard deviations of the roughness, harmonicity and mean pitch predictors are, respectively, 1, 1, and 0.52; see 'Discussion' for qualification of effect sizes)."

2) How are the priors defined?

We are not sure what is being asked for here. On p.10, we have “All population-level effects were given weakly informative priors (a Student’s t-distribution with a mean of 0, 3 degrees of freedom, and a scale of 1). This reflects our prior belief that the standardized effect size of each predictor is unlikely to be very large, and further serves to regularize (towards zero) any effects that may be only weakly informed by the data thereby decreasing the chance of false positives.”

3) Is not K>39 a really conservative threshold? I always understood K>sqrt(10) was roughly comparable to p<0.05. I think quantitatively transferring this to classical stats is complicated, since classical analyses can only measure p(data|null). However, assuming there are only two models in the analyses, wouldn't K>39 be the equivalent of p(null) = 0.025 and p(alternative) = 0.975?

For a Bayes factor, it would indeed be a very conservative threshold. But for an evidence ratio, a value of > 39 is comparable to a 2-sided p-value of < 0.05. Note that for a 2-way test, there need to be tail probabilities of 0.025 at both ends of the distribution to ensure the overall probability of being in either tail is 0.05. Hopefully this is now clear in the expanded p.10 explanation above.

4) Why is necessary to specify a range of values for which the effect is practically equivalent to zero? Shouldn't this depend on the variance of the estimates?

A range is required because the probability that a draw from a continuous probability distribution (such as the posterior distribution) is any exact value (such as 0) is precisely 0. In the above-quoted expanded p.10 explanation, we have now given the standard deviations of the predictors used in the model.

5) Since your two models (effect != 0, and effect=0) are mutually exclussive, I would expect the posteriors to sum up to one; why is this not the case? I can imagine why this may be a consequence of the choice of the interval [-.036,+.036]; however: is there not a better way to estimate the likelihood that the data is sampled from an effect distribution with zero mean, incorporating the empirical estimation of the variance?

As you say, the models are not mutually exclusive (the ROPE is not p(effect = 0), and the posterior probabilities are not p(effect != 0), the latter are p(effect is in the direction hypothesized)). We are aware of only two Bayesian techniques for testing the null, and the ROPE technique is standard and well-established and preferred by those who find Bayes factors conceptually and practically problematic.

Potential typos:
Second sentence in Methods: Participants from the Uruwa River Valley" reads a bit awkward; I would for instance use "participants were excluded".

This is changed to: “Interview data were missing from one of these participants, so they were removed from the analysis.”

Page 6, "The cadences and melodies blocks..." this sentence sounds a bit strange. Maybe you could write something like "because we measured ratings of ..."?

This is now changed to “The cadences and melodies blocks are not reported here because in those blocks we measured ratings of happiness, not finishedness; …”

Page 7, "When you hear which tune, which one is finished" is this correct?

Yes, this is correct. The literal translation of the sentence presented to participants in Nungon to English sounds a bit odd, but it is correct in their local language. We added the following to the manuscript to clarify: “(Note that this sentence sounds odd in English, but it follows the correct grammatical structural of Nungon).”

Page 8, "mdl_cut_int_triad_1" not listed in Table 5, maybe a typo?

Corrected.

Minor suggestions:
Tables could use self-contained explanatory captions.

The headings of several of the tables have been adapted and all should now be self-explanatory.

Tables and figure in the appendix could be numbered as A1, A2, ...

Done as requested.

Machine-print variable names and headings in Table 5 are really untransparent: I would rewrite then in human-readable format. Much less urgent, but maybe still worth consider, in the name of the variables in Table 7

The caption of Table 5 has been expanded to better explain the content, and the model names have been simplified a bit.
